# Supplementary material for: Chemical-induced colitis lowers mitochondrial bioenergetic function in colonic tissue with minimal impacts on the proteome
Source: Sci Rep. 2026 Jul 6;16:30833. doi: 10.1038/s41598-026-61224-z (PMC13634912; doi:10.1038/s41598-026-61224-z)
Supplement: Supplementary file 1 [file 41598_2026_61224_MOESM4_ESM.docx]

**Supplemental Table 1.** Proteomics Data. **A)** Exported Full Fasta results from Proteome discover 10.2.2. **B)** Analyzed Full Fasta results, including MEF calculation. **C)** Full Fasta heatmap of OxPhos proteins. **D)** Mouse MitoCarta 3.0 database. **E)** Exported results from Proteome Discover 10.2.2 using MitoCarta 3.0 database only. **F)** Analyzed results from MitoCarta 3.0 positive proteins **G)** Complex-specific protein and dehydrogenase ID key.

**Supplemental Table 2.** Source data. Individual data points for all figures.

**Supplemental Table 3.** Mouse bodyweight and wellness assessments throughout treatment duration.
